# Assessing the Elimination of User Fees for Delivery Services in Laos

**DOI:** 10.1371/journal.pone.0089784

**Published:** 2014-03-14

**Authors:** Chantelle Boudreaux, Phetdara Chanthala, Magnus Lindelow

**Affiliations:** 1 The Harvard School of Public Health, Department of Global Health and Population, Boston, Massachusetts, United States of America; 2 The World Bank, Human Development Sector, Vientiane, Laos; 3 The World Bank, Human Development Sector, Brasilia, Brazil; University of Alabama at Birmingham, United States of America

## Abstract

A pilot eliminating user fees associated with delivery at the point of services was introduced in two districts of Laos in March 2009. Following two years of implementation, an evaluation was conducted to assess the pilot impact, as well as to document the pilot design and implementation challenges. Study results show that, even in the presence of the substantial access and cultural barriers, user fees associated with delivery at health facilities act as a serious deterrent to care seeking behavior. We find a tripling of facility-based delivery rates in the intervention areas, compared to a 40% increase in the control areas. While findings from the control region suggest that facility-based delivery rates may be on the rise across the country, the substantially higher increase in the pilot areas highlight the impact of financial burden associated with facility-based delivery fees. These fees can play an important role in rapidly increasing the uptake of facility delivery to reach the national targets and, ultimately, to improve maternal and child health outcomes. The pilot achieved important gains while relying heavily on capacity and systems already in place. However, the high cost associated with monitoring and evaluation suggest broad-scale expansion of the pilot activities is likely to necessitate targeted capacity building initiatives, especially in areas with limited district level capacity to manage funds and deliver detailed and timely reports.

## Introduction

The means to reduce maternal mortality have been well described in the literature [1]. With hemorrhage, high blood pressure, infection, abortion and obstructed labor among the top causes of morbidity and mortality of women during pregnancy, ensuring access to skilled health professionals who can identify and effectively manage pregnancy-related risks is critical to increasing the chances of survival. As such, increasing skilled attendance at delivery has been an emphasis in many programs aiming to improve maternal health. Notably, this has been an important objective of the Millennium Development Goals (MDGs), the eight development goals that all UN member states have agreed to achieve by 2015. While several of the MDGs relate to the status and health of women, Goal 5 specifically targets maternal health and Goal 5a calls for a reduction of maternal mortality by three-quarters. Emphasizing the importance of attended delivery, the proportion of births attended by skilled health personnel is a key monitoring indicator for this goal [2].

According to a recent review, despite significant efforts throughout the world, only 13 countries are on track to attain their targets for MDG 5. The same review qualified progress in addressing maternal mortality as “slow” and “without any overall evidence of acceleration” [3]. Poor maternal health outcomes reflect the convergence of a number of factors, including sociocultural traditions around child bearing and delivery, health care quality and access to services [4]. Financial barriers are also believed to be an especially important constraint in many contexts. While the need to recover costs or a desire to rationalize use of services have led many countries to develop user fees systems over the years, there is concern that these fees are preventing use of services by those who most need them. Services such as antenatal and obstetric care are likely to be especially affected by the introduction of such fees; price responsiveness in the demand for health services is well documented, and is generally higher for preventive services than for curative care [5].

User fees for health care were introduced in dozens of low-income countries in the late 1980s and early 1990s [6]. These fees were argued to improve efficiency in the allocation of health services and increase the availability of financial resources available to facilities. It was further theorized that these funds could be used to improve the quality of care and even increase the equity of services [7]. However, their introduction resulted in substantial controversy and, over the last decade, numerous countries have moved to eliminate user fees, especially those associated with preventive and maternal care [8]–[12].

User fee elimination and exemption efforts have frequently been conducted as part of larger efforts aimed at addressing Millennium Development Goals 4 and 5–to reduce child mortality and improve maternal health, respectively. Increasing access to key services, such as skilled attendance at birth, has been a central part of many country's efforts to achieve these goals [13], and many countries have sought to eliminate these fees in an effort to increase health service utilization rates. However, studies examining the effects of user fees on maternal health services have seen mixed results. Researchers in Afghanistan found that, while antenatal visits increased immediately following the elimination of user fees, these effects were temporary, and no effect was found for institutional delivery [10]. While researchers in Ghana found a statistically significant increase in facility-based delivery following the removal of fees [14], researchers in South Africa found a statistically insignificant *decrease* in preventive services when user fees were abolished–potentially due to crowding out by an upswing in demand for curative service [11]. This latter case illustrates the complicated environment in which user fees exist and may hint at an underlying cause for the conflicting results. User fees do not alone determine health service utilization rates, but must interact with numerous cultural and environmental factors, including access to services, facility capacity and availability of resources, and socio-cultural norms around care seeking. Meanwhile, the political nature of the intervention may make collection of robust data a significant challenge. This is highlighted by a recent review, which identified substantial methodological issues surrounding the current evidence base related to the impact of fee exemptions on maternal health. In a systematic review of the literature, the authors found no randomized trials on the subject, and only one of the seventeen studies identified estimated the effects net of temporal trends [6].

While there is arguable growing consensus about the case for eliminating, or at least significantly reducing fees for key services, the implementation of user fee reform is often a challenge. Facilities or local administrative units that have relied on fee income to finance operations need to have this income substituted by other funding if fees are abolished, and the uncertain effect of fee removal makes it difficult to plan for human resource or supply needs [15]. This raises a number of administrative and logistical issues, not only related to the transfer of funds from federal or local government, but also to how to ensure adequate oversight in the use of funding and the delivery of services.

In this context, the Ministry of Health of Laos undertook a pilot study to assess the impact of user fees on local facility-based delivery rates, and whether eliminating them would successfully stimulate demand for services in a context of very low utilization and substantial non-financial barriers. This paper seeks to document the details of the pilot while using an interrupted time series analysis to assess its effect on facility-based delivery rates. An important goal of the pilot and associated evaluation was to learn lessons about implementation arrangements with a view to inform a possible national scale-up.

### Maternal Health Challenges, User Fees, and the “Free Delivery Pilot” in Laos

In 1996, with the Lao health sector facing severe strain and limited scope to increase the public budget, the government legalized user fees for specified procedures after more than 30 years of government-funded health services. A year later, fees were extended to create a Revolving Drug Fund (RDF) system at health facilities. These legislations would ultimately result in the widespread reliance upon user fees in government facilities throughout Laos [16]. With exceptions for vaccinations and some consultations, patients were, at the time that the pilot began, responsible for most medical fees. This included the costs associated with registration, tests, supplies, and any medicines needed.

User fees were introduced in a context of very low levels of utilization and high maternal mortality. In 1995, there were an estimated to be 970 maternal deaths per 100,000 live births [17]. Despite improvements over the past two decades, Laos continues to have among the highest maternal mortality rates in the region with the latest figures estimating 470 maternal deaths per 100,000 live births in 2010 Figure 1 [18]. Table 1 below provides an overview of key maternal health indicators for the country, highlighting the urban/rural disparities.

**Figure 1:**
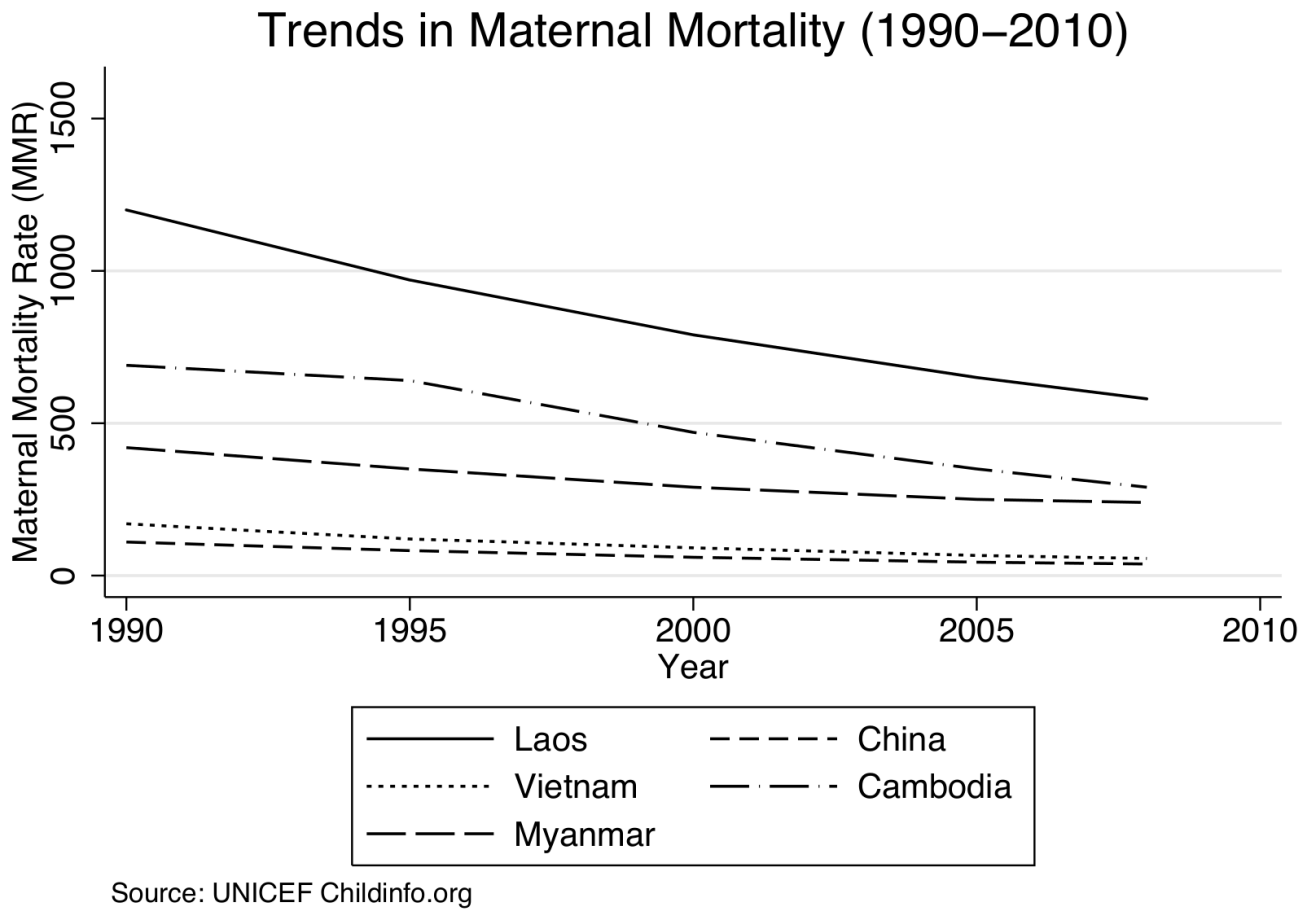
Trends in Maternal Mortality (1990–2010). (Source: UNICEF Childinfo.org).

**Table 1:** Maternal Health Indicators^1^.

	Urban	Rural	Rural no Roads
Infant Mortality Rate (IMR) per 1,000 live births	39	82	108
Under 5 Mortality Rate (U5MR) per 1,000 live births	45	94	136
Total Fertility Rate	2.2	3.4	4.8
Facility-Based Delivery (%)	74.2	29.2	11.6
Antenatal care (min. of 1 visit) (%)	85.7	51.7	20.5

1 Source: Lao Social Indicator Survey (LSIS) [28].

Poor maternal health outcomes in Laos reflect the convergence of a number of factors, including sociocultural traditions around child bearing [19], poor health care quality [20] and limited access [21]. Financial barriers associated with user fees are believed to exacerbate these constraints [22]. Surveys in southern Laos suggest highly variable costs associated with delivery. On average, women paid approximately 20 USD for uncomplicated deliveries conducted at health centers, 45 USD for deliveries at district hospitals, and 90 USD for those at provincial hospitals. Costs for deliveries by cesarean section vary even more – from an average of 70 USD in district hospitals to more than 550 USD in provincial hospitals [23].

By 2009, there was a growing consensus among government stakeholders and development partners that the small income from user fees did not justify the negative impact on the uptake of facility-based delivery services. However, there was limited evidence that the elimination–a logistically complex and expensive endeavor–would increase utilization rates in the Lao context [24]. A pilot was thus proposed to identify an effective implementation model and to quantify the effects on facility-based delivery rates. The pilot was launched in March 2009. Under the pilot, user fees associated with delivery were eliminated at health center and district hospitals in two districts of central Laos.

## Free Delivery Pilot

### Study Setting

The study was implemented in four districts in Savannakhet Province of central Laos. With a population of approximately 725,000 people, Savannakhet is the largest of the Laos' 16 provinces. Like most of Laos, Savannakhet is largely rural, sparsely populated and characterized by significant ethnic diversity. Infrastructure is poor year-round and seasonal floods between June and October completely isolate many areas during significant periods of the year. Of the total 139 districts in the country, 72 are considered “poor,” of which 47 are “high priority and poor.” The four districts covered by the study were all designated by the national government as high priority poor districts.

### Pilot Design

Pilot details were proposed by MOH officials and amended following a provincial consultation process. In establishing implementation details, stakeholders sought to balance minimal administrative complexity against the need for appropriate fiduciary controls. Given the country's widespread poverty and weak information systems, administrators opted for geographic targeting rather than the more expensive household level targeting. Ultimately, a two-tiered case-payment system with separate rates for complicated (25 USD) and normal (15 USD) deliveries was selected as a compromise between administrative complexity to the program and financial protection for health care providers. Similar concerns dominated the discussion of transportation payments, but with a different outcome. The substantial regional and seasonal variation in transportation costs compelled program managers to allow for reimbursement based on the actual fees charged by local taxi operators.

### Intervention

The Free Delivery Pilot was initiated in October 2009. In two of the four study districts, women were not charged any fees associated with delivering infants at health centers or hospitals. While both simple and complex delivery costs were included, post-delivery complications for the mother and child were not covered, nor were transportation costs from the woman's home to the local health center. In case of a complicated delivery, costs associated with referral to and from the district hospital for both the mother and one companion were covered, as were meal costs at the hospital. In the two control districts, women continued to pay for supplies used and a labor fee for delivery. In an effort to isolate the effect of financial constraints in the decision to seek care, no activities targeting health service quality were included in the study.

There is limited reach of media campaigns in Laos, and ensuring that local residents were aware of the pilot posed a particular challenge. Mobilization campaigns for the intervention were led by village elders, and supported by health workers during quarterly village outreach visits. Health staff led meetings in all catchment-area villages to ensure that pregnant women were aware of the pilot and of their rights to receive cost-free delivery cares at health centers.

**Figure 2:**
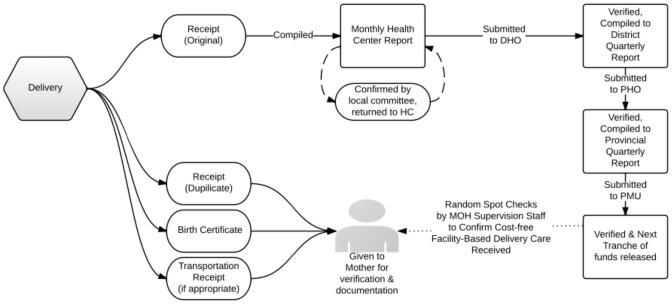
Reporting Flows for the Free Delivery Pilot.

Given weak fiduciary controls, establishing robust reimbursement systems was a critical concern for the pilot (Figure 2). The pilot introduced birth certificates, which were not in routine use across the country. Duplicate receipts for delivery were also drawn up – with one copy kept at the health center and the second given to the mother. Funding and reporting within the pilot followed a hierarchical model wherein quarterly financial transfers were made directly by the central government to the district health office, and the district accountant was responsible for financial management procedures. A preliminary tranche of funds was transferred prior to the initiation of the pilot, with additional funds transferred quarterly, contingent upon receipt of relevant reports.

The pilot relied upon local development committees, supplemented with random spot checks to villages by Ministry of Health staff. These committees are part of the local governance process, and hold monthly meetings to discuss development issues relevant to a cluster of villages 4–8 villages, with clusters generally aligning with the HC catchment areas. In the two districts, covering approximately 60,000 people, the pilot relied upon fourteen separate committees – one per health facility – to validate 133 delivery and expenditure reports for 2010. Administrative costs in the form of “sitting fees” were up to a third of the budget for the pilot and, while the committee structure is expensive from an administrative point of view, individual incentives small and meetings were often postponed, often resulting in serious delays in reporting and fund transfers to health center.

## Evaluation

### Ethical Review

This study received ethical review by the Internal Review Board of the Harvard School of Public Health.

### Study Design

**Figure 3:**
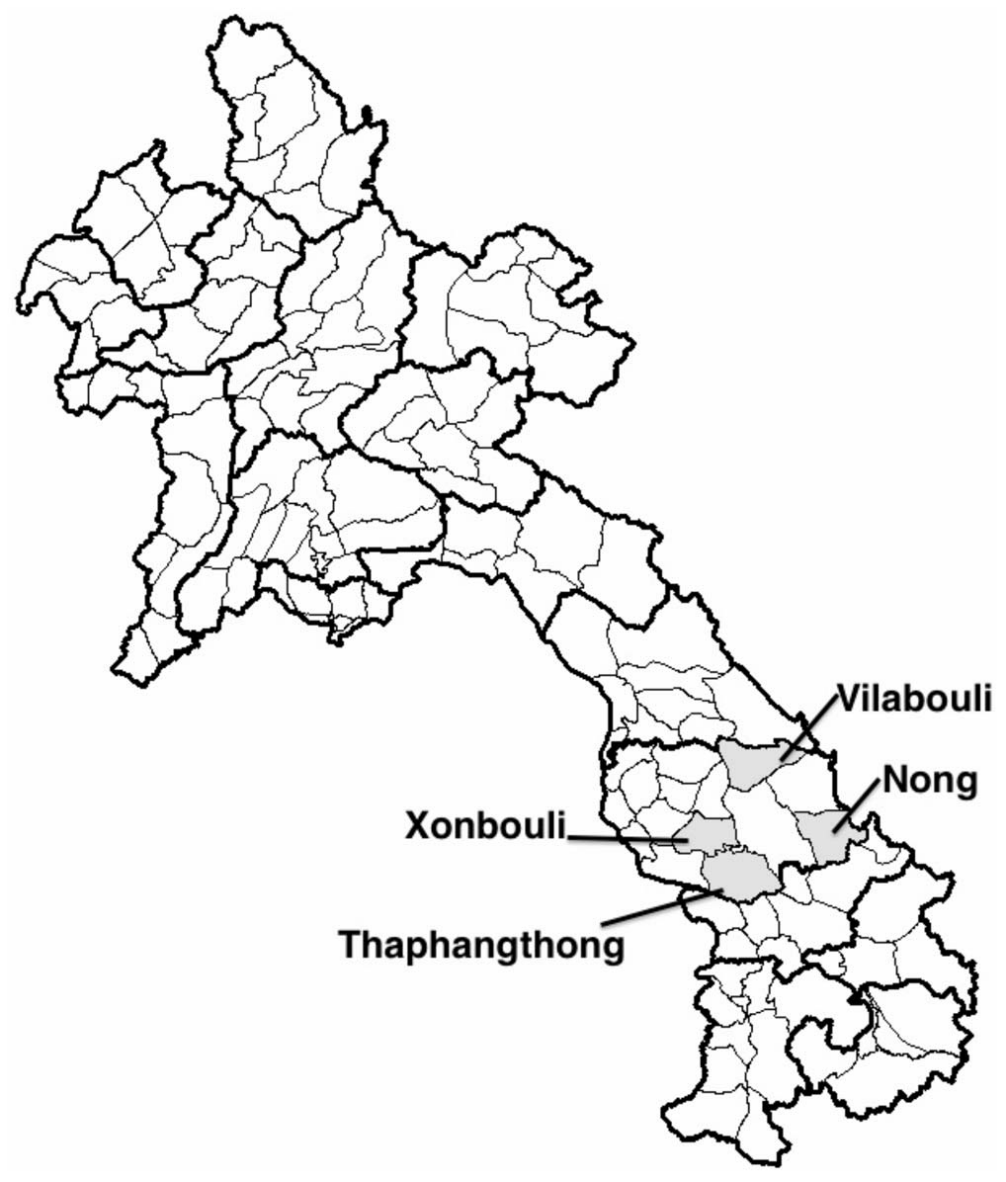
Map of Laos.

The study utilized an interrupted time series design with group assignment at the district level. Two districts (Nong and Thapangthong) were selected to receive the intervention. Similar districts in the same province (Xonbouli and Vilabouli) were selected to serve as controls (Figure 3). While the assignment of the pilot to districts was non-random, control districts were selected based on observable characteristics to ensure similarity with pilot districts along key dimensions, including high ethnic diversity, high poverty, and low levels of facility-based delivery. For analysis, routine data on facility-based delivery was gathered for the 24 months prior to the initiation of the pilot and 23 months following the initiation of the pilot to measure the changes associated with facility-based delivery. The study utilized aggregate data on deliveries from health facilities and hospitals by month. In case of potential outliers, districts were asked to follow up with health centers to confirm data accuracy. When districts confirmed that these outliers accurately reflected facility reports, they included in the data.

## Analysis

The pilot was initiated in October of 2009. Records on facility delivery were collected for the period of November 2007 through October 2011. To calculate coverage rates, estimates on the total number of births in each district were received from the Lao Department of Statistics. The outcome of interest was the percentage of deliveries taking place within health centers or district hospitals. Unconditional pre-post group mean differences are presented (Table 2).

**Table 2:** Assisted Delivery Rates in Study Districts.

	Pre-Intervention	Post-Intervention
Intervention Area	8.5%	23.7%
Non-Intervention Area	11.9%	18.4%
Difference	3.33	−5.27
T-test of difference	0.004	0.007

In order to facilitate coefficient interpretation, heterogeneity-adjusted linear probability models of the following functional form were estimated: 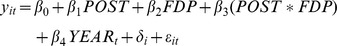

*y*_it_ is the percent of deliveries taking place at health facilities among woman in district i and period t, POST is a binary indicator, which equals 0 for baseline (*pre*: November 2007 – October 2009) observations and 1 for end line (*post*: November 2009 – October 2011) observations, and FDP is a binary indicator which equals 0 for control districts and 1 for intervention districts. The POST term captures the average improvements in both groups and the FDP captures the average difference between intervention and control districts at baseline, while the POST * FDP interaction term reflects the differences in changes across the two groups, and is the coefficient of interest in this analysis. τ captures time trends independent of the intervention. In order to capture locally time-invariant differences, district fixed effects δ are included in the model. In an alternative iteration of the model, the assumption of a linear time trend was dropped and the effect of time was captured through inclusion of monthly fixed effects. As all dependent variables are binary, Huber-White standard errors are applied to adjust for the non-normal distribution of the error terms. To address the spatial correlation of outcomes, standard errors are estimated taking into account clustering at district level.

All analysis was conducted using STATA 12 statistical software.

## Results

Population and health infrastructure characteristics for the districts are provided in Table 3 below. The population of the districts ranged from 23,300 in Nong District to nearly twice that in Xonbouli, and there were between 1,000 and 2,000 pregnancies per year in each district. Prior to the initiation of the pilot, deliveries were somewhat less likely to take place at medical facilities in the pilot districts than in control districts, but after two years of follow-up the pattern had reversed, with women living in the pilot areas more likely to seek professional care for delivery than those in control areas (Table 2).

**Table 3:** Descriptive Statistics.

	Population	% Living in Poverty	Expected births (per year)	Intervention Area	Number of Health Centers
Nong	23,300	28%	1,000	Yes	6
Thapangthong	34,750	19%	1,175	Yes	5
Xonbouli	55,650	17%	1,775	No	6
Vilabouli	33,475	20%	1,200	No	6

Table 4 provides an overview of statistical results from four models. The baseline model (1) shows the results of a simple difference-in-difference estimation. Skilled birth attendance was approximately 4.6 percentage points lower in intervention areas at the start of the study, and had increased by approximately 5.3 percentage points, overall, by the end of the study period. The pilot was associated with an additional 9.8 percentage point increase in skilled assistance at delivery.

**Table 4:** Statistical Results.

	(1)	(2)	(3)	(4)
Outcome variable: Coverage of Skilled Birth Attendance	Baseline Model	Baseline Model with Time Trends	Baseline Model with Time Trends & District Fixed Effects	Baseline Model with Month Fixed Effects
Regional Effect	−4.57	−4.57	−9.14**	–
	*(4.61)*	*(4.62)*	*(1.74)*	
Time Effect	5.30**	4.21	4.21	–
	*(0.94)*	*(4.96)*	*(4.96)*	
Pilot Effect	9.84*	9.84*	9.84*	10.96*
	*(3.53)*	*(3.54)*	*(3.56)*	*(4.32)*
Time Trend	–	0.05	0.05	–
		*(0.19)*	*(0.19)*	
District Fixed Effects	No	No	Yes	No
Month Fixed Effects	No	No	No	Yes
Constant	13.09*	12.51	18.65***	9.251*
	(4.60)	(6.05)	(2.22)	(3.26)
Observations	188	188	188	172
R-squared	0.306	0.307	0.499	0.648

“Regional Effect” shows the estimated difference between pilot and non-pilot areas, with non-pilot districts used as the baseline. “Time Effect” shows the estimated difference between pre- and post-treatment time periods. “Pilot Effect” gives the estimated impact of the pilot. “Time trend” assumes a linear time trend. “District Fixed Effects” include dummy variables for each of four districts. “Month Fixed Effects” include dummy variables for each of 47 months. Clustered, robust standard errors are in parentheses.

*** p<0.01,

** p<0.05,

* p<0.1.

Additional models suggest that the findings are robust to the model specification. Model (2) adds a linear time trend to the baseline model; results are not sensitive to this addition, with the pilot again associated with a 9.8 percentage point increase in skilled birth attendance. Model (3) adds both a linear time trend and district fixed effects. While this addition increases the overall R-squared of the model, it has no effect on the estimated effect of the pilot, which is, again, estimated at 9.8 percentage points. Model (4) drops the assumption of a linear time trend and includes monthly fixed effects. While the estimated impact of the pilot increases somewhat to approximately 11 percentage points, the results are not substantively different from those found in the baseline model.

**Figure 4:**
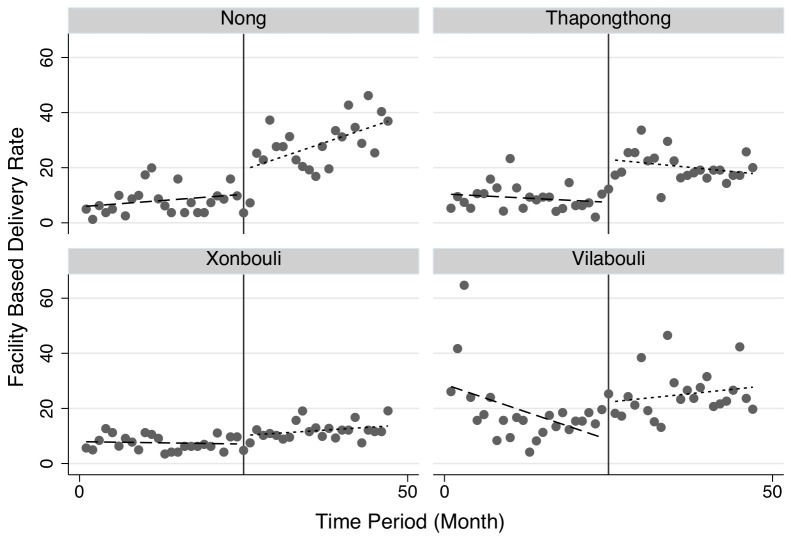
Facility-Based Deliveries in Pilot and Non-Pilot Districts.

**Figure 5:**
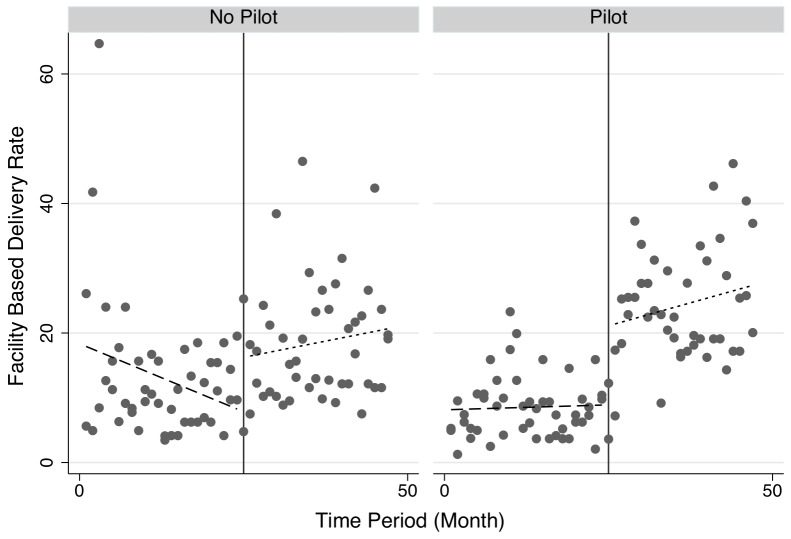
Facility Based Deliveries at Pilot (Nong and Thaphangthong) and Non-Pilot (Xonbouli and Vilabouli) Districts.

These results are shown graphically in Figures 4 and 5. Figure 4 shows separate trend lines by intervention status, pre- and post- initiation of the pilot. Figure 5 shows the same by district. The figures highlight an apparent jump in utilization at the time point at which user fees are eliminated in the 2 pilot districts of Nong and Thaphangthong, which is absent in the two control districts. The individual district view also allows a better view of the facility-based utilization rates in Vilabouli district – one of the two controls. As noted above, efforts were made to ensure data quality, and facilities were asked to confirm unexpected numbers. Nonetheless, the reported utilization rates in Vilabouli are more variable and, in a small number of cases, higher than expected. The monthly view of data shows that despite these few months of unexpectedly high utilization, the overall trend is of a gradual increase of facility-based deliveries in both of the control districts. This is in contrast to the large jumps in utilization rates in Nong and Thaphangthong noted above.

### Limitations

There are a number of limitations to this study. The study was small with few districts included. As shown in Figure 3, the pilot district of Thaphangthong borders on the control district of Xonbouli, which would increase the risk spill-over and tend to underestimate the true effect. Disaggregated data were unavailable beyond the district level and important individual level characteristics could not be accounted for. Due to the reliance on routine administrative data, information on survival and other outcome data were unavailable, and the presence of the study may have resulted in differential data quality during the study period. On this point, the data include a small number of potential outliers during the pre-pilot phase, and none in the post-pilot phase (Figure 4). However, if, as this data suggest, data are inflated during the pre-pilot period, this would underestimate of the true effect. Although the pilot occurred in during a period of intense outreach to increase skilled assistance at delivery, and we expect substantial mobilization in both intervention and control regions, the study is also unable to disentangle the effects of the outreach campaigns from those associated with the user fee elimination. Finally, the results rely on the assumption of common trends that underlies the empirical model.

## Discussion

The current study extends the knowledge base surrounding the effects of user fee removal on maternal health outcomes. Though largely consistent with findings in other countries, we find a larger overall effect size following the elimination of fees than do other researchers. A study of two national user fee exemption policies in Ghana found increases of 2.5%–7.5% following the introduction of policies aimed at eliminating user fees [25]. The only other study identified as having controlled for time trends took place in Afghanistan and found no effects. However, the authors note that deliveries were free in a large majority of facilities prior to the elimination of user fees [10]. The work is also consistent with a larger number of pre-/post- studies which are unable to account for underlying trends due to nation-wide roll out of the policies under investigation [12], [14], [26]. However, the substantial increase in utilization of services among control districts in our study highlights the importance of taking these time trends into account.

The rugged geography and limited infrastructure in Laos result in substantial difficulties in accessing delivery care. These difficulties are compounded by long-standing socio-cultural norms emphasizing traditional birthing practices. Nonetheless, the results of the pilot suggest that even in the presence of these access and cultural barriers, the high and unpredictable cost associated with delivery at health facilities acts as serious deterrent to seeking assisted care at facilities. The pilot found a tripling of facility-based delivery rates in the intervention areas, compared to a 40% increase of baseline rates in the control areas. The percentage of Lao women seeking skilled assistance at delivery had changed very little during the three decades prior to the initiation of the pilot. Our results suggest that this trend may at last be changing. Nonetheless, even with all fees removed, more than 75% of women delivered outside of a facility. While the increase in facility-based delivery rates identified in this pilot is promising, substantial work remains to be done. Work in other countries has highlighted the high cost of delivery care even in the face of fee exemptions. For example researchers in Tanzania found ongoing high out of pocket expenditures despite user fee exemptions. These were largely explained by the high cost of transportation and unofficial provider payments [27]. These issues are also likely to be important in Laos, as are the socio-cultural norms of many of the country's ethnic groups which emphasize home-based delivery [19].

The question remains whether alternative designs might be more effective. The pilot faced persistent delays in transferring necessary funds to health centers. While the reliance on existing committees for verification of activities and payments was expected to be a time- and cost-efficient decision, the aggregate number of small payments resulted in large costs overall even while small individual incentives were insufficient to encourage committee members to maintain a regular schedule. This resulted in delays in tranche transfers to health centers. Logistically, the committees rapidly became cumbersome, prone to delays and, in aggregate, expensive. Any attempts to scale the program would need to be done in the context of building adequate capacity at district level for both administration and service delivery.

Despite these challenges, the pilot is locally considered to be a success, and the Ministry of Health is considering a geographic expansion of its core activities. In Laos, as in many countries, attempts to make substantial changes to health care delivery are constrained by the existing capacity. The Free Delivery Pilot showed that immediate results could be gained on a small scale while using the existing structures.

## References

[1] Campbell OMR, Graham WJ (2006) Strategies for reducing maternal mortality: getting on with what works. Lancet 368: : 1284–1299. Available: http://www.ncbi.nlm.nih.gov/pubmed/17027735. Accessed 5 November 2012. 10.1016/S0140-6736(06)69381-1 17027735

[2] The Millenium Development Goals (2013) The Millennium Development Goals Report 2013.

[3] Lozano R, Wang H, Foreman KJ, Rajaratnam JK, Naghavi M, et al. (2011) Progress towards Millennium Development Goals 4 and 5 on maternal and child mortality: an updated systematic analysis. Lancet 378: : 1139–1165. Available: http://www.ncbi.nlm.nih.gov/pubmed/21937100. Accessed 12 December 2013. 10.1016/S0140-6736(11)61337-8 21937100

[4] Ronsmans C, Graham WJ (2006) Maternal mortality: who, when, where, and why. Lancet 368: : 1189–1200. Available: http://www.ncbi.nlm.nih.gov/pubmed/17011946. Accessed 11 December 2013. 10.1016/S0140-6736(06)69380-X 17011946

[5] Cohen J, Dupas P (2008) Free distribution or cost-sharing? evidence from a malaria prevention experiment. Available: http://www.nber.org/papers/w14406. Accessed 23 December 2013.

[6] Dzakpasu S, Powell-Jackson T, Campbell OMR (2013) Impact of user fees on maternal health service utilization and related health outcomes: a systematic review. Health Policy Plan. Available: http://www.ncbi.nlm.nih.gov/pubmed/23372035. Accessed 11 December 2013. 10.1093/heapol/czs142 23372035

[7] Akin J, Birdsall N, Ferranti D De (1987) Financing health services in developing countries: an agenda for reform. Available: http://scholar.google.com/scholar?hl=en&btnG=Search&q=intitle:Financing+Health+Services+in+developing+countries#0. Accessed 11 December 2013.

[8] Asante F, Chikwama C, Daniels A, Armar-Klemesu M (2007) Evaluating the economic outcomes of the policy of fee exemption for maternal delivery care in ghana. Ghana Med J 41: 110–117 Available: http://www.pubmedcentral.nih.gov/articlerender.fcgi?artid=2279082&tool=pmcentrez&rendertype=abstract. 18470328 10.4314/gmj.v41i3.55277 PMC2279082

[9] Deininger K (2005) Economic and Welfare Impact of the Abolition of Health User Fees: Evidence from Uganda. J Afr Econ 14: : 55–91. Available: http://jae.oupjournals.org/cgi/doi/10.1093/jae/ejh034. Accessed 11 December 2013.

[10] Steinhardt LC, Aman I, Pakzad I, Kumar B, Singh LP, et al. (2011) Removing user fees for basic health services: a pilot study and national roll-out in Afghanistan. Health Policy Plan 26 Suppl 2: : ii92–103. Available: http://www.pubmedcentral.nih.gov/articlerender.fcgi?artid=3247786&tool=pmcentrez&rendertype=abstract. Accessed 11 December 2013. 10.1093/heapol/czr069 PMC3247786 22027924

[11] Wilkinson D, Gouws E, Sach M, Karim SS (2001) Effect of removing user fees on attendance for curative and preventive primary health care services in rural South Africa. Bull World Health Organ 79: 665–671 Available: http://www.pubmedcentral.nih.gov/articlerender.fcgi?artid=2566476&tool=pmcentrez&rendertype=abstract. 11477970 PMC2566476

[12] Witter S, Khadka S, Nath H, Tiwari S (2011) The national free delivery policy in Nepal: early evidence of its effects on health facilities. Health Policy Plan 26 Suppl 2: : ii84–91. Available: http://www.ncbi.nlm.nih.gov/pubmed/22027923. Accessed 11 December 2013. 10.1093/heapol/czr066 22027923

[13] Campbell OMR, Graham WJ (2006) Strategies for reducing maternal mortality: getting on with what works. Lancet 368: : 1284–1299. Available: http://www.ncbi.nlm.nih.gov/pubmed/17027735. Accessed 5 November 2012. 10.1016/S0140-6736(06)69381-1 17027735

[14] Penfold S, Harrison E, Bell J, Fitzmaurice ANN (2007) Evaluation of the Delivery Fee Exemption Policy in Ghana: Population estimates of changes in delivery service utilization in two regions. 41: 100–109. PMC2279083 18470327

[15] Ridde V, Morestin F (2011) A scoping review of the literature on the abolition of user fees in health care services in Africa. Health Policy Plan 26: : 1–11. Available: http://www.ncbi.nlm.nih.gov/pubmed/20547653. Accessed 14 December 2013. 10.1093/heapol/czq021 20547653

[16] Thome JM, Pholsena S (2009) Lao People's Democratic Republic: Health Financing Reform and Challenges in Expanding the Current Social Protection Schemes. Promoting sustainable strategies to improve access to health care in the Asian and Pacific Region. Bangkok. pp. 71–102.

[17] Department of Economic and Social Affairs (2011) Millenium Development Goals Indicators. United Nations. Available: http://mdgs.un.org/unsd/mdg/Data.aspx. Accessed 12 December 2011.

[18] The World Bank (2013) World DataBank.

[19] Sychareun V, Hansana V, Somphet V, Xayavong S, Phengsavanh A, et al. (2012) Reasons rural Laotians choose home deliveries over delivery at health facilities: a qualitative study. BMC Pregnancy Childbirth 12: : 86. Available: http://www.pubmedcentral.nih.gov/articlerender.fcgi?artid=3449206&tool=pmcentrez&rendertype=abstract. Accessed 8 December 2012. 10.1186/1471-2393-12-86 PMC3449206 22925107

[20] Manithip C, Edin K, Sihavong A, Wahlström R, Wessel H (2012) Poor quality of antenatal care services-Is lack of competence and support the reason? An observational and interview study in rural areas of Lao PDR. Midwifery. Available: http://www.ncbi.nlm.nih.gov/pubmed/22776568. Accessed 8 December 2012. 10.1016/j.midw.2011.12.010 22776568

[21] Douangphachanh X, Ali M, Outavong P, Alongkon P, Sing M, et al (2010) Availability and use of emergency obstetric care services in public hospitals in Laos PDR: a systems analysis. Biosci Trends 4: 318–324 Available: http://www.ncbi.nlm.nih.gov/pubmed/21248430. 21248430

[22] Douangvichit D, Liabsuetrakul T, McNeil E (2012) Health care expenditure for hospital-based delivery care in Lao PDR. BMC Res Notes 5: : 30. Available: http://www.pubmedcentral.nih.gov/articlerender.fcgi?artid=3295644&tool=pmcentrez&rendertype=abstract. Accessed 8 December 2012. 10.1186/1756-0500-5-30 PMC3295644 22243656

[23] The World Bank (2013) Maternal Health Out-of-Pocket Expenditure and Service Readiness in Lao PDR:

[24] Lagarde M (2008) The impact of user fees on health service utilization in low- and middle-income countries: how strong is the evidence? Bull World Health Organ 86: : 839–848. Available: http://www.who.int/bulletin/volumes/86/11/07-049197.pdf. Accessed 12 December 2013. 10.2471/BLT.07.049197 PMC2649541 19030689

[25] Dzakpasu S, Soremekun S, Manu A, Ten Asbroek G, Tawiah C, et al. (2012) Impact of free delivery care on health facility delivery and insurance coverage in Ghana's Brong Ahafo Region. PLoS One 7: : e49430. Available: http://www.pubmedcentral.nih.gov/articlerender.fcgi?artid=3500286&tool=pmcentrez&rendertype=abstract. Accessed 12 December 2013. 10.1371/journal.pone.0049430 PMC3500286 23173061

[26] Ridde V, Richard F, Bicaba A, Queuille L, Conombo G (2011) The national subsidy for deliveries and emergency obstetric care in Burkina Faso. Health Policy Plan 26 Suppl 2: : ii30–40. Available: http://www.ncbi.nlm.nih.gov/pubmed/22027917. Accessed 11 December 2013. 10.1093/heapol/czr060 22027917

[27] Kruk ME, Paczkowski M, Mbaruku G, de Pinho H, Galea S (2009) Women's preferences for place of delivery in rural Tanzania: a population-based discrete choice experiment. Am J Public Health 99: : 1666–1672. Available: http://www.pubmedcentral.nih.gov/articlerender.fcgi?artid=2724466&tool=pmcentrez&rendertype=abstract. Accessed 2 November 2012. 10.2105/AJPH.2008.146209 PMC2724466 19608959

[28] Ministry of Health and Lao Statistics Bureau and and ICF International (2012) Lao Social Indicator Study (LSIS).

